# Glucose Fluctuations in Acute Ischemic Stroke

**DOI:** 10.7759/cureus.61939

**Published:** 2024-06-08

**Authors:** Antigoni Fountouki, Thomas Tegos, Elizabeth Psoma, Keli Makedou, Nikolaos Kakaletsis, Georgia Kaiafa, Triantafyllos Didangelos, Dimitrios Theofanidis, Christos Savopoulos

**Affiliations:** 1 Department of Nursing, International Hellenic University, Thessaloniki, GRC; 2 1st Department of Neurology, AHEPA University General Hospital, School of Medicine, Aristotle University, Thessaloniki, GRC; 3 Department of Radiology, AHEPA University General Hospital, Aristotle University, Thessaloniki, GRC; 4 Laboratory of Biochemistry, AHEPA University General Hospital, School of Medicine, Aristotle University, Thessaloniki, GRC; 5 1st Propaedeutic Department of Internal Medicine, AHEPA University General Hospital, Aristotle University, Thessaloniki, GRC; 6 1st Propaedeutic Department of Internal Medicine, AHEPA University General Hospital, School of Medicine, Aristotle University, Thessaloniki, GRC; 7 1st Propaedeutic Department of Internal Medicine/Diabetic Care Unit, AHEPA University General Hospital, School of Medicine, Aristotle University, Thessaloniki, GRC

**Keywords:** oxfordshire community stroke project, infarct topography, cgm, hyperglycemia, stroke

## Abstract

Introduction: The Oxfordshire Community Stroke Project denotes four subtypes of ischemic stroke (total and partial anterior infarct, posterior, and lacunar). Hyperglycemia has been associated with a larger infarct size and poor prognosis.

Aim: The purpose of the study was to investigate the correlation of glucose fluctuations with the Oxford sub-categories and patient outcomes using a blinded continuous glucose monitoring system.

Methods: This is a non-interventional prospective observational study. Stroke patients with symptoms onset in the last 24h, participated in the study. A glucose sensor was placed for 72 hours. Disability was assessed using the modified Rankin Scale. Stroke subtypes were compared with total mean glucose and time in range using ANOVA analysis. Multiple ordinal logistic regression was employed to analyze outcomes and survival.

Results: The sample consisted of 105 diabetic and non-diabetic patients. The overall mean glucose was 127.06 mg/dL and the time in range (70-140 mg/dL) was 70.98%. There was no significant difference between the stroke sub-categories and the total mean glucose. For every one-point increase in the time in range, we expect a 1.5% reduction in the odds of having a worse outcome. Patients with total anterior infarct are 2.31 times more likely to have a worse outcome than lacunar patients.

Conclusion: The utilization of the Oxford classification may not be necessary for managing acute ischemic stroke glucose levels. Achieving glucose regulation and an increase in time in range can be attained through meticulous control, potentially extending life expectancy. Continuous glucose monitors may aid in achieving this objective.

## Introduction

Stroke affects approximately 13.7 million people globally with an annual mortality rate of about 5.5 million [1]. The Oxfordshire Community Stroke Project denotes four subtypes of ischemic stroke: a) Total anterior circulation infarct - TACI, b) partial anterior circulation infarct - PACI, c) posterior circulation infarct - POCI, and d) lacunar stroke - LACI [2].

TACI constitutes around 17%-21% of all strokes [3]. It is a severe type of stroke associated with higher mortality rates, raised healthcare costs, long hospital stays [4], and a significant rate of depression [5].

All three of the following need to be present for a diagnosis of a TACI: Unilateral weakness (and/or sensory deficit) of the face, arm, and leg, homonymous hemianopia, and higher cerebral dysfunction. A PACI is a less severe form of TACI, in which only part of the anterior circulation has been compromised [6]. Wood et al. [3], reported that in a sample of 675 patients with TACI, within 30 days, 40% died, 56% had severe disabilities and only 4% remained independent.

POCI accounts for approximately 20%-25% of all cases of acute ischemic stroke (AIS) [7] and involves damage to the area of the brain supplied by the posterior circulation. Up to 35% of strokes in these patients may remain undiagnosed. An unrecognized posterior stroke on initial assessment is linked to an eight-fold increased risk of death [8].

Lacunar infarcts represent approximately 25% of ischemic strokes and are usually associated with a more favorable prognosis. A LACI is a subcortical stroke that occurs secondary to small vessel disease [9]. There is no loss of higher cerebral functions [6].

Glucose fluctuations in AIS patients are common. It is reported that two-thirds of patients admitted with stroke present with hyperglycemia, regardless of pre-existing diabetes or not [10], due to dysregulation of pre-existing diabetes as well as stress hyperglycemia [11]. Stress hyperglycemia in hospitalized patients has been defined as a plasma glucose concentration >140 mg/dL without evidence of previous diabetes [12].

Scott et al. [13], studied admission glucose in 303 stroke patients classifying them according to Oxford topography. There were no statistically significant differences between stroke subtypes in terms of age, sex, or prevalence of diabetes mellitus (DM). However, patients with TACI had admission hyperglycemia greater than 108 mg/dL and a higher mean admission glucose than the other subtypes. More than 50% of patients in each subtype had a glucose value >108 mg/dL at admission.

Hyperglycemia is detrimental to the ischemic brain. Baird et al. [14], using a continuous glucose monitoring system (CGM) in 24 stroke patients suggested that persistent hyperglycemia is an independent determinant for the expansion of cerebral infarction and is associated with a worse functional outcome in stroke patients.

Diabetic patients have a poorer survival and recovery expectancy after a stroke compared to non-diabetics [15]. Capes et al. [16] concluded that in patients without a history of DM with AIS, even a moderate increase in glucose levels significantly increases mortality and the risk of poor neurological recovery.

Wada et al. [17] examined the relationship between glucose parameters and clinical findings in AIS patients by including 100 stroke patients. They applied glucose monitoring for the first 72 hours. They observed that high glucose levels and time percentages above 144 mg/dL during the initial 72 hours following AIS were associated with death or significant dependence at 3 months after the initial episode.

In patients with AIS, current guidelines recommend maintaining plasma glucose levels in the range of 140-180 mg/dL. Nukui et al. [18] demonstrated that many AIS patients have plasma glucose levels outside the recommended range in the acute stroke phase.

Allport et al. [19] confirmed hyperglycemia in 81% of diabetic and 32% of non-diabetic patients during admission. Non-diabetic patients experienced a 50% glucose increase eight hours after symptom onset. A significant relationship was found between hyperglycemia duration and glucose levels on entry. In the diabetic group, hyperglycemia was observed in the first eight hours, while non-diabetic patients experienced a decrease in glucose values. The rate of hyperglycemia increased from 58% to 78% between 48 and 88 hours.

In a Greek study by Palaiodimou et al. [20], the researchers included 62 ischemic and hemorrhagic stroke patients in total and found no correlation of glycemic variability with patient outcomes at three months.

This study aimed to determine whether glucose fluctuations are associated with infarct topography according to Oxfordshire classification and patient outcomes after AIS for a period of up to six months after discharge, using a CGM. The Oxford classification is based on clinical manifestations. If glucose variation differs between subgroups, this could be a predictor of potential glycemic variation and should be taken into account in the care plan even if AIS is not detected in the first CT scan.

## Materials and methods

Participants

This is a non-interventional prospective observational study that did not modify the clinical management of the patients and studied whether glucose fluctuations are related to the topography and outcome of AIS. The study included patients who were admitted to the First Department of Propaedeutic and Internal Medicine in AHEPA University General Hospital, Aristotle University of Thessaloniki, during the period 2020-2022.

Inclusion criteria were patients presenting to the Emergency Department within the first 24 hours after onset of symptoms, men and women, diabetics and non-diabetics as well as patients with a previous stroke with a modified Rankin Scale (mRS) ≤2 participated in the study.

Exclusion criteria were fever (> 37ºC), previous AIS with modified Rankin Scale > 2, a transient ischemic attack, cancer within the last five years, and eligibility for thrombolysis or thrombectomy.

Prior to data collection, permission was granted from the Bioethics Committee of the Aristotle University of Thessaloniki (29/7/2020/6.261).

Procedure and ethical considerations

All patients in the sample, before giving written consent, were primarily screened for dementia using the Mini-Mental State Examination Test. In cases of patients with impaired levels of consciousness, a first-degree relative was asked to grant consent, following the provisions of the Code of Medical Ethics.

Continuous glucose monitoring was performed with Medtronic's blinded Envision Pro system (Medtronic, Dublin, Ireland) for 72 hours after admission. During hospitalization, glucose management was performed in all patients as in standard clinical care. The modified Rankin scale was used to assess disability levels. Measurements took place on the day of admission and discharge and at the first-, third-, and sixth-month post-discharge. All patients underwent a CT scan at the same CT Scanner (Somatom Emotion, Siemens Medical Solutions USA, Inc., Malvern, PA). In cases where the infarct was not visualized on the first CT scan, the procedure was repeated after 24 hours. Patients were divided into the four subtypes (TACI, PACI, POCI, LACI).

Statistical analysis

For descriptive statistical analysis, continuous variables are expressed as mean ± SD, while discrete variables are expressed as absolute and relative frequencies. To test normality, the “Normal Q-Q plot,” “Detrended Normal Q-Q plot,” and “Box Plot” were employed. Relationships between a quantitative variable and a dichotomous variable were conducted using the Student's t-test or the Mann-Whitney U test, depending on normality. Analysis of variance (ANOVA) or the Kruskal-Wallis test was used, depending on whether the assumption of normality was satisfied. Categorical variables were analyzed using the x2 test.

Moreover, multiple ordinal logistic regression was used to investigate the factors predicting ordinal variables and to control for confounding factors. To determine the factors affecting survival, Cox Regression was used. Statistical processing of the data was performed using SPSS 26 software (IBM Corp., Armonk, NY). The minimum value of statistical significance level was set at 5%.

## Results

Demographic and clinical characteristics

The study sample consisted of 105 patients, 61 women (58.1%) and 44 men (41.9%), with an average age of 80.95 years. 43 (41%) patients suffered from Diabetes Mellitus (DM). On admission, the mean serum glucose was 146.14mg/dL, and the mean glycosylated hemoglobin (HbA1c) was 5.80%. Based on the Oxford categorization patients were divided as follows: TACI = 25 patients (23.8%), PACI = 32 (30.5%), POCI = 23 (21.9%), and LACI = 25 (23.8%).

The total mean glucose (TMG), glycemic variability (Coefficient of Variation - CV), and the percentage of time in range (TIR - 70-140 mg/dL) were calculated to be 127.06 mg/dL, 13.06%, and 70.98%, respectively. Table 1 depicts the sample's descriptive characteristics.

**Table 1:** Descriptive characteristics Data are numbers (%) for categorical variables, mean ± SD for continuous variables, CV: coefficient of variation, LACI: lacunar infarct, OCSP: Oxfordshire Community Stroke Project, PACI: partial anterior circulation infarct, POCI: posterior circulation infarct, TACI: total anterior circulation infarct, TIR: time in range, TMG: total mean glucose.

Descriptive characteristics		Values
Sex (male)		44 (41.9%)
Diabetes mellitus		43 (41.0%)
OCSP classification	TACI	25 (23.8%)
	PACI	32 (30.5%)
	POCI	23 (21.9%)
	LACI	25 (23.8%)
Age (years)		81.0 ± 6.9
Admission HbA1c (%)		5.8 ± 1.0
Admission Glucose (mg/dL)		146.1 ± 55.1
TMG in all stroke patients (mg/dL)		127.1 ± 29.8
TMG in non-diabetics (mg/dL)		112.2 ± 19.0
TMG in diabetics (mg/dL)		148.6 ± 29.5
CV (%)		13.0 ± 6.0
TIR (70-140 mg/dL)		71.0 ± 32.6

Infarct topography and TMG

Descriptive measures of TMG in the first three days after diagnosis of Ischemic Stroke in each Oxford sub-type, and overall, are shown in Table 2.

**Table 2:** Infarction location and total mean glucose (TMG, mg/dL) levels CI: Confidence Interval, LACI: lacunar infarct, OCSP: Oxfordshire Community Stroke Project, PACI: partial anterior circulation infarct, POCI: posterior circulation infarct, SD: standard deviation, TACI: total anterior circulation infarct.

OCSP classification	Min-max	Mean (SD)	95% CI
TACI (n=25)	89-194	131.5 (28.5)	119.8-143.3
PACI (n=32)	86-212	126.3 (28.3)	116.1-136.5
POCI (n=23)	68-194	126.2 (32.7)	112.0-140.3
LACI (n=25)	83-236	124.4 (31.5)	111.4-137.4
Total (n=105)	68-236	127.1 (29.8)	121.3-132.8

**Figure 1:**
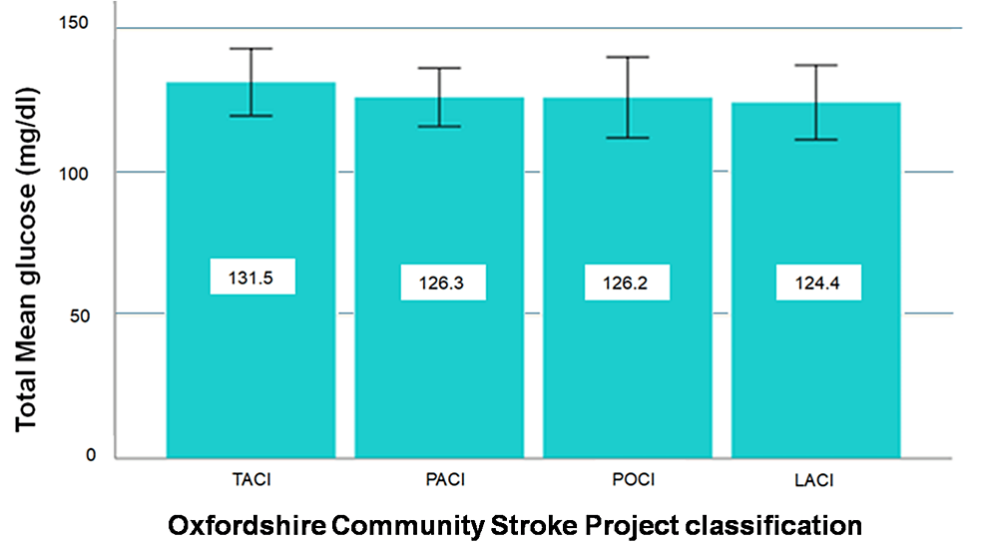
Total mean glucose (TMG) levels according to infarction location TACI: total anterior circulation infarct, PACI: partial anterior circulation infarct, POCI: posterior circulation infarct LACI: lacunar infarct. The error bars represent 95% confidence interval.

“Normal Q-Q plot,” “Detrended Normal Q-Q plot,” and “Box Plot” graphs show that the data come from a normal distribution. Based on the result of Levene's homogeneity test (F(3, 101)=0.135, p=0.939), we assume that the hypothesis of homoscedasticity is valid. Therefore, the analysis was performed by ANOVA test. The ANOVA test did not reveal a significant difference in the mean value of the TMG between the categories of the Oxford classification (F(3, 101)=0.260, p=0.854) (Figure 1).

Infarct topography and TIR

ANOVA test revealed no significant difference in the mean value of glucose TIR (70-140 mg/dL) in the first three days after diagnosis of ischemic stroke between the categories of Oxford Classification (F(3, 101)=0.640, p=0.591).

**Figure 2:**
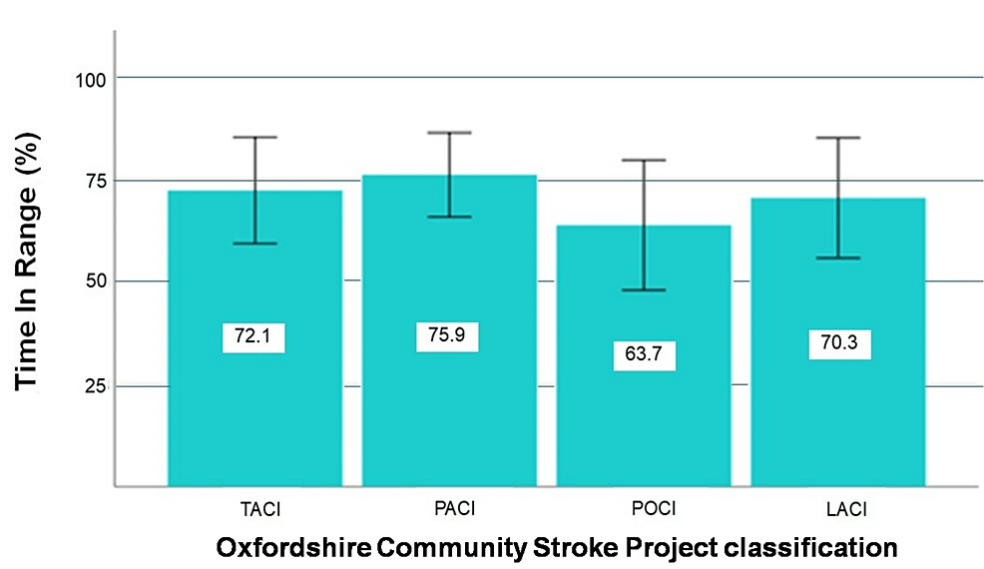
Time in range (TIR) according to infarction location TACI: total anterior circulation infarct, PACI: partial anterior circulation infarct, POCI: posterior circulation infarct LACI: lacunar infarct. The error bars represent 95% confidence interval.

As far as glucose on admission is concerned, based on the Mann-Whitney U statistical test, patients with PACI had on average a higher glucose concentration on admission than patients with POCI. (p-value=0.007). Figure 2 depicts the TIR (70-140 mg/dL) in the first 72 hours after diagnosis of AIS.

Glucose variation and outcomes for up to six months after stroke

Data analysis was performed using an ordinal logistic regression model with the dependent variable being patient outcome for up to six months post-discharge. The categories of the dependent variable of outcome were a) movement independence (mRS 0-1), b) moderate (mRS 2-3) to severe disability (mRS 4-5), and c) death (6).

The independent variables under investigation are glycemic variability (GV), the TIR, the TMG, and the additional potential confounding factors: a) biological sex (male/ female), b) age, c) DM, d) admission HbA1C, e) admission glucose, and f) Oxford Ischemic Stroke category (TACI/ PACI/ POCI/ LACI).

The null hypothesis of equal slopes for all logits in the analysis is not rejected (p = 0.180), so Ordinal Logistic Regression was applied. The results show that (when all other variables in the model are held constant): For each year increase in age, for those >65, we expect a 7.4% (95% CI: 2.9%-12.1%, p=0.001) increase in the odds of having a worse outcome. For every one-point increase in TIR (70-140 mg/dL) in the first three days after AIS diagnosis, we expect a 1.5% (95% CI: 0.3%-2.7%, p=0.018) reduction in the odds of having a worse outcome. Moreover, TACI patients are 2.31 (95% CI: 0.18%-8.3%, p=0.023) times more likely to have a worse outcome than LACI patients.

Likelihood of survival in the first six months after discharge

We investigated the survival of 79 patients discharged from hospital. The sample of patients consisted of 47 women (59.5%) and 32 men (40.5%), mean age 80.15 years of which 49 (62%) did not have DM. On admission, their mean blood glucose was 145.13 mg/dL and their HbA1c was 5.79%.

The overall mean glucose, GV, and percentage of TIR (70-140 mg/dL) were calculated 126.76 mg/dL, 12.81%, and 72.96%, respectively.

At the end of the six-month follow-up period, survival was checked. Survival analysis was performed using a Cox subsample with nine independent variables (biological sex, age, DM, HbA1c, admission blood glucose, TMG, GV, and TIR).

We observe that biological sex, age, and TIR (independent variables) are those that explain, statistically significantly, the overall survival time, of patients discharged from the hospital. Specifically female patients have 64.1% lower risk of dying than male patients (95% CI: 0.3%-87.1%, p=0.049). For every year increase in age, >65 years, we expect a 16% (95% CI: 5.9%-27.4%, p=0.001) increase in the risk of death. Furthermore, for every unit increase in TIR (70-140 mg/dL), we expect a 3.5% (95% CI: 0.8%-6.1%, p=0.013) reduction in the risk of death.

## Discussion

We performed a 72-hour continuous glucose monitoring, taking into account the four subtypes of the Oxford classification, which is based on the manifestation of symptoms and signs and has been evaluated as easy to apply, having good interobserver reliability and ability to predict prognosis. To ensure greater reliability and to exclude transient attacks, the topography was confirmed with a CT scan. The glucose sensor was placed in all patients in the upper arm. No irritation, hematoma, or allergy was observed in any patient.

In Greek hospitals, stroke patients ≥ 65 are admitted to an internal medical ward and those <65 are admitted to a neurology ward. For this reason, the study sample has an average age of 80.95 years.

The TMG of the three-day monitoring of all patients was 127.06 mg/dL. Based on the analysis, there was no statistically significant difference in the TMG between the AIS study groups. The literature search did not identify any studies published so far investigating the correlation of glucose levels during the acute phase of stroke using a CGM system, between the sub-groups of the Oxford classification in order to make comparisons.

The Oxford classification of AIS mainly gives information about the infarct topography. However, a TACI is of greater clinical importance with a poorer prognosis than a PACI or a LACI. If hyperglycemia is a manifestation of the size of the infarction as often reported in the literature, a difference in the GV between these stroke subgroups would be expected. This may be due to the glucose management of the patients during their hospitalization as the admission glucose was higher (146.14 mg/dL±55.05) than the TMG of the three-day monitoring (127.06 mg/dL±29.79).

On admission, mean plasma glucose was 146.14 mg/dL (non-diabetics 125.04 mg/dL and diabetics 176.55 mg/dL). From the review of the literature, it is found that plasma glucose can show elevated values ​​during the admission of patients with AIS. The percentages of hyperglycemic stroke patients on admission range from 50% to 75%, although this percentage varies among researchers. The wide range in the incidence of hyperglycemia may also be due to the different glucose lower limits as set by different authors [13,15,21].

Considering 120 mg/dL as a hyperglycemic threshold, in the sample of the present study 67% of patients had hyperglycemia on admission. The threshold of 140 mg/dL identifies 48 patients as hyperglycemic, i.e., 46% of the sample.

TIR is another important parameter for estimating normoglycemia. The findings in the present study are similar to the literature [18], as patients' TIR was on average 70.98%. TIR did not differ between AIS subgroups.

In this study, we found that for every unit increase in the percentage of time within the TIR (70-140 mg/dL) as set by the authors, in the first three days after the diagnosis of AIS, we estimate a 1.5% decrease in the likelihood of having a worse outcome. For each unit increase in the percentage of time within the target range (70-140 mg/dL), in the first 72 hours post-AIS onset, we expect a 3.5% reduction in the risk of death.

The outcome of the patients in the present study was assessed by applying the Modified Rankin Scale. Patients were assessed on the first day after admission, at discharge, and in the first, third, and sixth months. Regarding the result by topographical categories of AIS, it has been found that in this particular sample, patients with TACI had a 2.31 times higher risk of a worse outcome than patients with LACI.

Patient mortality was increased in general and in each of the examined categories. According to the literature, TACI has a death rate of 56% at 6 months and a mortality/disability of 96% (mRS:4-5). In the current study, the TACI group had a mortality of 72% and a total mortality/disability incidence (mRS:4-5) equal to 88%.

Kammersgaard et al. [22] found that very old age significantly predicts short- and long-term prognosis in AIS. Sharma et al. (12) found that elderly patients had a worse prognosis than younger patients, despite having the same CT characteristics. We found that for every year older than 65, there is a 7.4% increase in worse outcomes and a 16% increase in mortality risk. The COVID-19 quarantine period negatively impacted stroke recovery and survival, with delays in medical attention, changes in emergency department functioning, patient relocation, house restrictions, and lack of rehabilitation treatment all linked to poorer prognosis.

GV refers to changes in plasma glucose levels, often caused by impaired or absent glycemic autoregulation or a rapid decline in insulin. Intermittent exposure to high plasma glucose has been found to have a deleterious effect, and recent clinical data show that GV increases the risk of microvascular and macrovascular complications and mortality in patients with diabetes, regardless of glycated hemoglobin level. The mean GV in the study was 13.06%, and no significant difference was found between diabetics and non-diabetics or between AIS topography subgroups.

The current study holds some limitations with the most profound one being that the study did not examine other patients' disorders or biochemical markers. Yet, the sample size of the current study is among the largest in the literature. characteristics.

## Conclusions

Our findings indicate that the Oxford classification is unnecessary in the management of AIS glucose levels. Glucose regulation and a rise in TIR can be achieved with careful control to prolong life expectancy. CGMs can help reach this goal.

Admission glucose values ​​appear to decrease during hospitalization. The individualized glucose management of patients may complicate the study of hyperglycemia following AIS. Despite the fact that hyperglycemia is assessed in the literature by glucose values, a combination of management, number of measurements, medication, and the necessity of corrective interventions may be required in order to assess the hyperglycemic tendency of patients.
